# Recombinant Human IFNα-2b Response Promotes Vaginal Epithelial Cells Defense against *Candida albicans*

**DOI:** 10.3389/fmicb.2017.00697

**Published:** 2017-04-20

**Authors:** Ting Li, Xiaoxi Niu, Xu Zhang, Suxia Wang, Zhaohui Liu

**Affiliations:** ^1^Department of Obstetrics and Gynecology, Peking University First HospitalBeijing, China; ^2^Laboratory of Electron Microscopy, Ultrastructural Pathology Center, Peking University First HospitalBeijing, China

**Keywords:** vulvovaginal candidiasis, vaginal epithelial cells, *Candida albicans*, rhIFNα-2b, cytokines

## Abstract

Classical antifungal drugs have been subjected to restrictions due to drug toxicity, drug resistance, bioavailability, and detrimental drug interactions. Type I interferon (IFN) exerts direct distinct immunostimulatory or immunomodulatory actions; however, little is known regarding the anti-fungal reactions of vaginal epithelial cells (VECs) induced by the type I IFN response. Therefore, in the present study, we evaluated the cytotoxic activity, immunocompetent cytokine responses, and non-B IgG production of the VK2/E6E7 VEC line following recombinant human IFN α-2b (rhIFNα-2b) treatment in response to *Candida albicans*. When treated with rhIFNα-2b, the production of IL-2, IL-4, and IL-17 were significantly up-regulated compared to the infected control cells (*P* < 0.05). Our scanning electron microscopy results revealed that *C. albicans* can invade VECs by inducing both endocytosis and active penetration. RhIFNα-2b was able to transform the VECs into a thallus and stretched pattern, promoting the fusion of filopodia to form a lamellipodium and enhancing the mobility and the repair capacity of the VECs. In addition, rhIFNα-2b could effectively inhibit the adhesion, hyphal formation, and proliferation of *C. albicans*. Collectively, these responses restored the immune function of the infected VECs against *C. albicans in vitro*, providing a theoretical basis for this novel treatment strategy.

## Introduction

*Candida albicans* is an opportunistic fungal pathogen that typically exists as a harmless commensal in the urogenital tract; however, approximately 75% of women who are of reproductive-age suffer from a vaginal Candida infection at least once in their lifetime (Sobel, 1997; Brown et al., 2012). In addition to the cost of treatment, these symptomatic episodes cause a significant decrease in the quality of life (McClelland et al., 2009).

Previous studies have demonstrated that soluble immune modulators (e.g., cytokines and chemokines) play putative roles for local host defense and immunotherapeutic strategies to control or prevent infections with *C. albicans*. Interleukin-2 (IL-2) both mediates the proliferation and activation of T cells, as well as activates natural killer cells and B cells (Borish and Steinke, 2003). IL-4 signaling and subsequent cytokine production (e.g., IL-10) in response to Candida are known to dampen cell-mediated protection, leading to increased susceptibility to Candida infection (Romani, 1999). In addition, IL-6 is a multifunctional cytokine with both pro-inflammatory and immunoregulatory functions (Basu et al., 2008). IL-8 is a well-known mediator of neutrophil recruitment to areas of inflammation and/or infection *in vivo* (Baggiolini and Clark-Lewis, 1992). Moreover, the level of IL-8 is a useful biomarker of the antimicrobial response in the female genital tract (Spear et al., 2008). While IL-17 plays a role in host defense against multiple infectious diseases, it also promotes inflammatory-mediated pathology in autoimmune diseases and other conditions (Onishi and Gaffen, 2010). Moreover, there is accumulating evidence for the protective role of the Th17-axis against Candida infection in which IL-17 signaling promotes the production of antimicrobial peptides and chemotactic mediators (e.g., S100 proteins) by epithelial cells (Liang et al., 2006). Recently, there is growing evidence that many normal non-B cells, including certain gland epithelial cells or endothelial cells, can also express immunoglobulin G (IgG), the function of which remains unclear (Jiang et al., 2015). Moreover, gland epithelial cell-derived IgM was reported to exhibit natural antibody activity (Hu et al., 2012). Based on these findings, we proposed that IgG may be expressed by VECs and provide antimicrobial activity in response to Candida infection.

Classical antifungal drugs have been subjected to restrictions due to drug toxicity, drug resistance, bioavailability, and detrimental drug interactions (Brown et al., 2012). Moreover, improvements in the clinical outcome of VVC in the last decade have experienced little progress (Smeekens et al., 2013). Thus, deciphering the various molecular and cellular mechanisms of human antifungal immunity for the development of new drugs to treat fungal infections is an urgent issue (Armstrong-James and Harrison, 2012; Leibund Gut-Landmann et al., 2012). Recent emphasis has shifted to adjuvant immunotherapy to further reduce the morbidity of VVC, and previous studies have supported the prominent role of the type I IFN pathway in the anti-Candida host defense response (Smeekens et al., 2013).

The type I IFN family includes a singular IFN-β and multiple IFN-α subtypes (Biondo et al., 2011). Although the antiviral and antitumor functions of IFN-α/β have been well established, a growing number of recent studies have focused on the role of type I IFN as part of the anti-inflammatory response (Biondo et al., 2011). Moreover, type I IFN exerts direct distinct immunostimulatory or immunomodulatory actions in response to extracellular stimuli of viral or bacterial origin following TLR engagement, including proinflammatory and microbicidal responses against common extracellular bacterial pathogens (Zimmerer et al., 2008). In addition, a number of studies have demonstrated the effectiveness of this family of cytokines in reducing inflammation in various experimental and clinical settings for the management of autoimmune or inflammatory disorders, including MS, FMF, and Behcet’s syndrome (Kötter et al., 2004; Barkhof et al., 2007; Tweezer-Zaks et al., 2008). However, little is known about the activation of the type I IFN system and the induction of anti-fungal responses in VECs, which are considered the first line of defense provided by the innate immune system (Cole, 2006).

Therefore, the aim of this study was to evaluate the mechanisms underlying the antifungal effects of type I IFNα in VVC for the wider clinical application as a potential antifungal drug for the prevention of VVC.

## Materials and Methods

### Materials

Recombinant human IFNα-2b (molecular weight: 17 Kda) was obtained from ZhaoKe (Hefei) (Pharmaceutical Co., Ltd., Hefei, China). The specific activity of 1.0 × 10^5^ U/g of rhIFNα-2b was 5 g in a transparent hydrophilic gel. A 5 g hydrogel (water soluble) was dissolved in a 50 mL serum-free RPMI1640 culture medium to prepare a drug stock solution of 100 mg/mL, and was passed through a 0.22 μM membrane filter for sterilization. All drug solutions were stored at -20°C until further experiments. All other reagents and chemicals were of the highest purity grade available.

*Candida albicans* strains (ATCC-64548) were grown aerobically overnight on SDA (Becton Dickinson, Cockeysville, MD, USA) plates, were propagated in YPD medium, and incubated overnight in an orbital shaker at 37°C. For *in vitro* experiments, *C. albicans* cells were harvested and washed in sterile PBS (Sigma Chemical Co., St. Louis, MO, USA) and the cell suspension was prepared in RPMI 1640 and adjusted to a cell density of 1.0 × 10^5^ cells/mL for all experiments. All experiments were performed in triplicate on three separate occasions.

### Cell Line and Culture

Human VEC line, VK2/E6E7 cells (ATCC^^®^^ CRL-2616), were obtained from the American Type Culture Collection (Rockville, MD, USA) and grown cultured in K-SFM (Gibco, USA) supplemented with 5 ng/mL recombinant epidermal growth factor and 50 μg/mL bovine pituitary extract (Invitrogen Corporation, Grand Island, NY, USA) with 100 units/mL each of penicillin and streptomycin (Life Technologies, Grand Island, NY, USA) at 37°C with 5% CO_2_ in a high humidity environment. All experiments were performed with VK2 cells during the exponential growth phase (48 h after plating).

### Cell Treatment

The VK2 cells were cocultured with *C. albicans* (1 × 10^5^/mL) at a ratio of 1:1 in separate wells in 24-well tissue culture plates (Costar, Corning, NY, USA) in a humidified atmosphere containing 5% CO_2_ at 37°C for 12 h. Following a coculture for 12 h, the culture medium was replaced with 1.25 mg/mL of rhIFNα-2b (IC_10_) as described above for an additional 24 h.

### Cytotoxic Assay

Cell viability was determined by an *in vitro* CCK-8 (Dojindo Laboratories, Tokyo, Japan) assay as previously described (Wang et al., 2016). Briefly, the cells were plated into 96-well plates (2 × 10^4^ cells/well) into a humidified atmosphere containing 5% CO_2_ at 37°C for 24 h before the cells were treated. After 24 h, the DMEM medium was removed and replaced with concentrations of 0, 1.25, 2.5, 5, and 10 mg/mL rhIFNα-2b for 24 h at 37°C. At the end of the incubation, the VK2 cells were treated with a CCK-8 solution (10 μL in 100 μL K-SFM) and incubated at 37°C for 1 h. The absorbance was recorded at 450 nm using a micro-plate reader (BioTek, Winooski, VT, USA). Cell viability (%) was calculated according to the following formula: Cell viability % = [OD_450_ (treated) - OD_450_ (blank)]/[OD_450_ (control) - OD_450_ (blank)] × 100.

### Enzyme-linked Immunosorbent Assay (ELISA)

The collected supernatants were centrifuged (1500 × *g* for 10 min at 4°C) prior to measuring the concentrations of bioactive functional factors. Only the supernatant was separated and stored in a deep freezer at -80°C for later analysis. The IL-2, IL-4, IL-6, IL-8, IL-17, as well as the epithelial-derived IgG and sIgA concentrations were determined in the supernatant samples using an ELISA Ready-SET-Go kit (eBioscience, USA) according to the manufacturer’s instructions. The concentrations of lubricin less than minimal sensitivity were considered to be 0 pg/mL. The absorbances were read with a 490 nm filter using a Ceres 900 automated microplate reader (Bio-Tek Corp., Winooski, VT, USA), with bovine serum albumin used as the standard.

### Scanning Electron Microscopy (SEM)

The cell samples were prepared for imaging by adding a freshly prepared fixative buffer (2.5% glutaraldehyde in 0.1 M sodium cacodylate buffer) overnight at 4°C. The cells were centrifuged at 12,000 × *g* and the pellet was cut into small blocks (∼2 mm squares) with an acetone-cleaned razor blade. The samples were rinsed twice (3 min each) in 0.1 M phosphate buffer and then placed in 1% Zetterquist’s osmium for 30 min. The samples were subsequently dehydrated through graded ethyl alcohol (70% ethanol for 10 min, 95% ethanol for 10 min, 100% ethanol for 20 min) and dried using the critical point drying method (BALTEC, Balzers, Liechtenstein). The dried samples were glued onto SEM stubs, sputter-coated with a 10 nm thick layer of gold (BALTEC, Balzers, Liechtenstein), viewed and photographed using a scanning electron microscope (S-3400N, Hitachi, Japan) in a high-vacuum mode at 15 kV. The number of living VK2 cells, dead VK2 cells, hypha, and blastospores were evaluated by counting 30 consecutive fields under SEM (magnification × 2000).

### Statistical Analysis

Data are presented as the means ± SEM. The statistical analyses were performed using SPSS version 13.0 (SPSS, Chicago, IL, USA). A ANOVA was used to analyze the differences for the multiple comparisons as appropriate. Whenever statistically significant differences were found, LSD *post hoc* test was used. Each experiment was performed in triplicate and three independent experiments were performed. A threshold value of *P* < 0.05 was considered to be statistically significant.

## Results

### Effects of rhIFNα-2b on VK2/E6E7 Cell Viability

**FIGURE 1:**
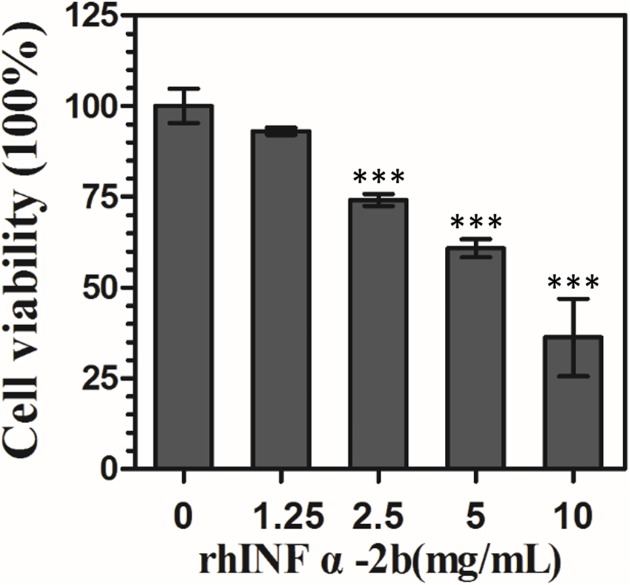
**Effect of rhIFNα-2b on VEC viability**. VK2/E6E7 cells were treated with rhIFNα-2b for 24 h. The mean values for the remaining four values and standard deviations (error bars) are shown. ^∗∗∗^, significant difference compared to 0 mg/mL of rhIFNα-2b control group (*P* < 0.0001).

To mimic the clinical situations in which antibiotics or antifungals may be safe and well-tolerated in the human body, rhIFNα-2b was used to treat VK2 cultures for 24 h to determine whether the concentrations of 0, 1.25, 2.5, 5, and 10 mg/mL of rhIFNα-2b would adversely affect VEC growth (**Figure 1**). As shown in **Figure 1**, after 24 h treatment, high doses (>1.25 mg/mL) of rhIFNα-2b did have a significant inhibitory effect on VEC viability and growth, while there were no significant effects observed in the presence of low doses (0 and 1.25 mg/mL). IC_10_ as calculated by SPSS 13.0 was 1.25 mg/mL. This dose was defined as a safe dose with little toxic side effects and the highest concentration at which there were no effects of rhIFNα-2b on cell viability (≥90% survival) (Namiecinski et al., 2004; Qiao et al., 2013). Thus, 24 h treatment with a dose of rhIFNα-2b up to 1.25 mg/mL exhibited no cytotoxic effect on the VK2 cells, and was selected for further experiments.

### rhIFNα-2b Modulates Cytokine Release by Human VECs

**FIGURE 2:**
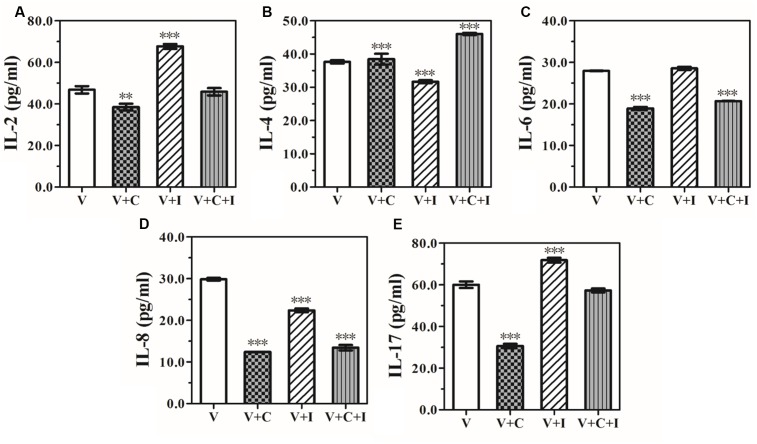
**Effect of rhIFNα-2b on the production of IL-2 (A)**, IL-4 **(B)**, IL-6 **(C)**, IL-8 **(D)**, and IL-17 **(E)** (expressed as pg/mL) by the VEC line, VK2/E6E7 cells cultivated alone, grown with 1.25 mg/mL rhIFNα-2b, and infected with *C. albicans* (1 × 10^5^/mL). The supernatants were collected and the cytokine levels were assessed by performing an ELISA 12 h post-infection and a subsequent 24 h of co-incubation with 1.25 mg/mL of rhIFNα-2b. V represents the VECs cultivated alone; V+I represents VECs co-incubated with 1.25 mg/mL of rhIFNα-2b for 24 h; V+C represents VECs infected with *Candida albicans* for 12 h; V+C+I represents the VECs infected with *C. albicans* for 12 h, then treated with 1.25 mg/mL of rhIFNα-2b for another 24 h. ^∗∗^, significant difference compared to the V group (*P* < 0.001); ^∗∗∗^, significant difference compared to the V group (*P* < 0.0001).

The level of IL-2, IL-4, IL-6, IL-8, and IL-17 production by VK2 cells was determined after 12 h of challenge with *C. albicans*. All of the cytokines listed above were significantly down-regulated compared to the control group (IL-2: *P* = 0.006; other ILs: *P* < 0.0001). In particular, IL-2 and IL-17 declined 0.82-fold from baseline levels of 46.81 ± 3.07 pg/mL (*P* = 0.006) and 0.51-fold from 60.03 ± 2.71 pg/mL (*P* < 0.0001), respectively (**Figure 2**). When treated with 1.25 mg/mL rhIFNα-2b alone, the level of IL-2 (67.67 ± 1.99 pg/mL, *P* < 0.0001), and IL-17 (71.84 ± 1.92 pg/mL, *P* < 0.0001) were significantly up-regulated, while the level of IL-4 (31.70 ± 0.78 pg/mL, *P* < 0.0001), and IL-8 (22.37 ± 0.75 pg/mL, *P* < 0.0001) were significantly down-regulated. Moreover, when rhIFNα-2b was added 12 h post-challenge with *C. albicans*, IL-2 and IL-17 were significantly up-regulated when compared with the untreated vaginal cells infected with *C. albicans* (45.87 ± 3.04 pg/mL, *P* = 0.011 and 57.32 ± 1.63 pg/mL, *P* < 0.0001, respectively). In addition, the level of IL-4 production exhibited significant up-regulation (46.04 ± 0.62 pg/mL, *P* < 0.0001) compared to the untreated vaginal cells infected with *C. albicans* (**Figure 2**). We determined the Th1/Th2 balance by calculating the IL-2/IL-4 ratio (Netea et al., 2002, **Table 1**).

**Table 1:** Th1/Th2 cytokine ratios.

Cytokine (pg/mL)	V	V+I	V+C	V+C+I	*F*-value	*P*-value
IL-2	46.81 ± 3.07	67.67 ± 1.99	38.48 ± 2.84	45.87 ± 3.04	61.519	<0.0001^∗^
IL-4	37.65 ± 0.85	31.70 ± 0.78	23.12 ± 0.76	46.03 ± 0.62	485.992	<0.0001^∗^
IL2/IL-4	1.24 ± 0.08	2.14 ± 0.09	1.66 ± 0.07	1.00 ± 0.08	117.183	<0.0001^∗^

*V represents the VECs cultivated alone; V+I represents the VECs co-incubated with 1.25 mg/mL of rhIFNα-2b for 24 h; V+C represents the VECs infected with *Candida albicans* for 12 h; V+C+I represents the VECs infected with *C. albicans* for 12 h, then treated with 1.25 mg/mL of rhIFNα-2b for another 24 h. ^∗^*P* < 0.0001*.

### The Regulation Pattern of VEC-derived IgG by rhIFNα-2b

**FIGURE 3:**
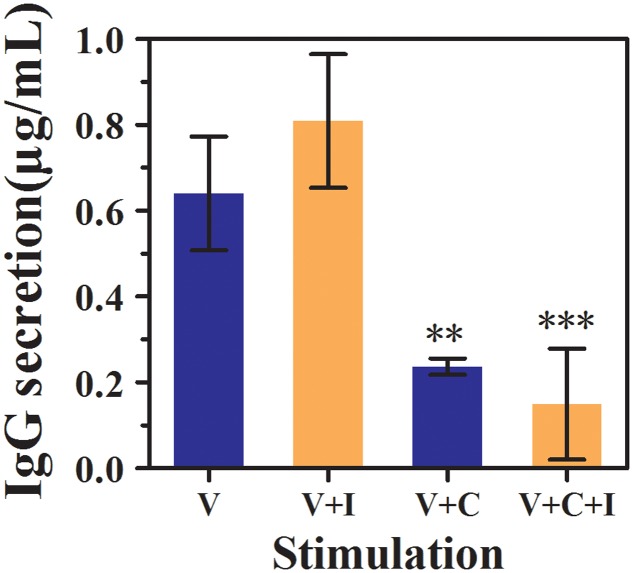
**Effect of rhIFNα-2b on the production of vaginal epithelial-derived IgG (expressed in μg/mL) by the VEC line, VK2/E6E7 cells**. Supernatants were collected, and the IgG levels were assessed by performing an ELISA after 12 h of infection and a subsequent 24 h of co-incubation with rhIFNα-2b. V represents the VECs cultivated alone; V+I represents the VECs co-incubated with 1.25 mg/mL of rhIFNα-2b for 24 h; V+C represents the VECs infected with *C. albicans* for 12 h; V+C+I represents the VECs infected with *C. albicans* for 12 h, then treated with 1.25 mg/mL of rhIFNα-2b for another 24 h. ^∗∗∗^, significant difference compared to the V group (*P* < 0.0001); ^∗∗^, significant difference compared to the V+C group (*P* = 0.004). Each sample was repeated three times. The error bars indicate the standard deviation.

To determine whether RhIFNα-2b had an effect on IgG and sIgA secretion by VECs, the production of epithelial-derived IgG and sIgA was compared between the treated and untreated vaginal cells infected with *C. albicans* (**Figure 3**). However, sIgA previously assumed to be the most abundant Ig isotype secreted by VECs was undetectable in our study (Data not shown).

Surprisingly, we found that VECs spontaneously secrete epithelial-derived IgG under baseline conditions (**Figure 3**). The baseline level of IgG secreted by the VK2 cells was 0.64 ± 0.13 μg/mL, which increased to 0.81 ± 0.16 μg/mL when the cells were treated with rhIFNα-2b alone (*P* = 0.126) and dropped sharply to 0.24 ± 0.02 μg/mL following *C. albicans* infection (*P* = 0.004). The level of IgG was found to change minimally following the co-inoculation of rhIFNα-2b following infection compared to the untreated vaginal cells infected with *C. albicans* (0.24 ± 0.02 vs. 0.15 ± 0.13, *P* = 0.402).

### rhIFNα-2b Promotes the Repair of Infected Cells

**FIGURE 4:**
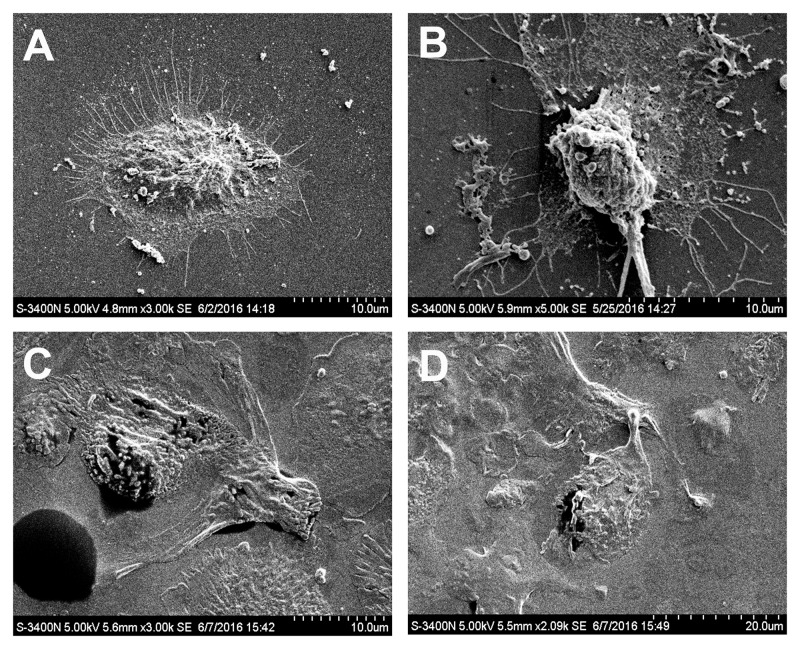
**Effect of rhIFNα-2b on VEC-mediated anti-Candida activity**. SEM of the control cells **(A)**, *C. albicans* infected cells at 12 h **(B)**, and rhIFNα-2b treated cells **(C,D)**. **(C,D)** represent the VECs infected with *C. albicans* for 12 h, then treated with 1.25 mg/mL rhIFNα-2b for another 24 h. Microvilli are indicated by small red arrows, filopodia are indicated with a small yellow arrow, pseudohyphae are indicated with a small blue arrow, and living VK2 cells indicated with white arrows.

**FIGURE 5:**
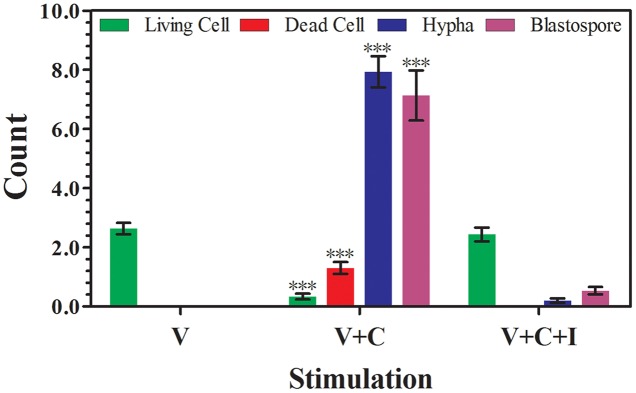
**Effect of rhIFNα-2b on VECs and *C. albicans***. The number of living cells, dead cells, hypha, and blastospores were evaluated by counting 30 consecutive fields under SEM (magnification × 2000) after 12 h of infection and a subsequent 24 h of co-incubation with rhIFNα-2b. V represents the VECs cultivated alone; V+C represents the VECs infected with *C. albicans* for 12 h; V+C+I represents the VECs infected with *C. albicans* for 12 h, then treated with 1.25 mg/mL of rhIFNα-2b for another 24 h. ^∗∗∗^, significant difference compared to the V group and V+C+I (*P* < 0.0001). The error bars indicate the standard deviation.

We examined the interactions between rhIFNα-2b on *C. albicans*-infected VECs using SEM. Microvilli are actin-based structures found on the apical aspect of many epithelial cells, and differ in function and characteristic morphology based on type (Sauvanet et al., 2015). The VK2 cells were covered with microvilli or microvilli crests (net-like membrane ruffles; **Figure 4A**). After 12 h of infection, large amounts of blastoconidia adhered to the surface of the infected cells and germinated to produce pseudohyphae penetrating into the VK2 cells (**Figure 4B**). Significant differences were noted in the number of living VK2 cells, dead VK2 cells, pseudohyphae, and blastospores after 12 h of infection under SEM (magnification × 2000) compared with the uninfected condition (*P* < 0.0001; **Figure 5** and **Table 2**).

**Table 2:** Quantification of VK2 cells, hypha, and blastospores (SEM, magnification × 2000).

Counts	V	V+C	V+C+I	*F*-value	*P*-value
**Living cells**	2.63 ± 0.189	0.33 ± 0.100	2.43 ± 1.278	48.700	<0.0001^∗^
**Dead cells**	0.00 ± 0.000	1.30 ± 0.199	0.00 ± 0.000	42.866	<0.0001^∗^
**Hypha**	0.00 ± 0.000	7.93 ± 0.527	0.20 ± 0.074	216.528	<0.0001^∗^
**Blastospores**	0.00 ± 0.000	7.13 ± 0.848	0.53 ± 0.124	64.494	<0.0001^∗^

*V represents the VECs cultivated alone; V+C represents the VECs infected with *C. albicans* for 12 h; V+C+I represents the VECs infected with *C. albicans* for 12 h, then treated with 1.25 mg/mL of rhIFNα-2b for another 24 h. ^∗^*P* < 0.0001*.

However, rhIFNα-2b treatment significantly reduced the number of invasive blastoconidia and pseudohyphae (**Figures 4C,D**). Moreover, rhIFNα-2b could both effectively inhibit the adhesion, hyphal formation, and proliferation of *C. albicans*, but could also notably restore VEC morphology and viability. Significant differences were noted in the number of living VK2 cells, dead VK2 cells, pseudohyphae, and blastospores after rhIFNα-2b treatment under SEM (magnification × 2000) compared with the infected condition (*P* < 0.0001; **Figure 5** and **Table 2**). In contrast, there was no significant difference between the uninfected and treated conditions (*P* = 0.441, *P* = 1.000, *P* = 0.647, and *P* = 0.448, respectively).

Pseudopodia, including filopodia and lamellipodium, are specialized actin subcellular structures which mediate cellular adhesion, migration, and transmigration through cell and matrix barriers (Schachtner et al., 2013). The treated adherent epithelial cells gradually assumed a more thallus-like morphology and completely stretched, maintaining an intact cell membrane, and exhibiting filopodia fusion to produce a lamellipodium. *C. albicans* is capable of stimulating epithelial cells to produce pseudopod-like structures that surround the fungus and draw it into the cell in a process that may be one of the mechanisms used for epithelial cell invasion (Phan et al., 2007). Furthermore, the initially observed invasive blastoconidia and pseudohyphae were significantly reduced or completely absent in these treated VECs.

## Discussion

According to different sources and antigenicity, IFN can be divided into three distinct types: IFNα, IFNβ, and INFγ. IFNα is primarily derived from T cells, B cells, NK cells, and macrophages, is also called leukocyte IFN, and belongs to the family of type I IFNs. Currently, human IFNα is one the first IFNs produced by recombinant DNA technology, and has been extensively demonstrated to regulate the immune system and induce a potent innate immune response against various types of infectious diseases (Tian et al., 2013; McNab et al., 2015).

Due to its immunotherapeutic effect against viral infection, IFNα has been synthetically produced in different forms as a therapeutic drug, including rhIFNα-2b in a hydrophilic gel (Syed and Ahmadpour, 1998). Increasing evidence shows that for external use, this novel immune regulating agent can:(1) induce the production of endogenous IFNs; thus, regulating immune function, enhancing the phagocytic function of macrophages, and effectively improving the local vaginal immunity (Smeekens et al., 2013); (2) promote the synthesis of antiviral proteins which play a vital role in mediating antiviral effects (Zhao et al., 2016); (3) inhibit tumor cell proliferation and the suppression of oncogenes, inducing cellular apoptosis through mitochondria-, ER stress-, and death receptor-induced apoptotic pathways (Shi et al., 2016); and (4) exert estrogen-like effects via estrogen receptor β subtypes (Niu et al., 2015), thereby improving the micro-environment of the vagina and promoting the regeneration of the squamous epithelium. VVC is most commonly observed in individuals with immune defects, a compromised immune system, or following the use of estrogen analogous to immunocompromised settings (De Repentigny, 2004; Naglik et al., 2008). Therefore, the application of adjunctive immunotherapy to improve host defense is an attractive strategy to improve the outcome of patients with VVC (Delsing et al., 2014).

The balance between Th1 and Th2 cytokines is important during the initiation of an immune response against *C. albicans*. In particular, a Th1 cytokine response is associated with resistance to candidiasis, whereas a Th2 response results in promoting a humoral, proinflammatory response associated with increased susceptibility to infection (Netea et al., 2002). In addition to Th1 and Th2 cell responses, the role of the Th17 subset in host defense against fungi has become increasingly evident (Hernández-Santos and Gaffen, 2012). Moreover, Th17 cells that produce IL-17 are crucial for protection against oral or mucocutaneous candidiasis (Curtis and Way, 2009).

According to our results, when VK2/E6E7 cells were infected with *C. albicans*, the production of all cytokines, especially IL-17, was significantly dampened. However, following treatment with rhIFNα-2b, the level of all cytokines (i.e., IL-2, IL-4, and IL-17) was significantly increased compared to the infected control. When treated with rhIFNα-2b alone, an increase in the balance of the Th1/Th2 ratio reflected the enhanced cell-mediated protection against *C. albicans* with a Th1-biased response. Our results indicate that rhIFNα-2b can indirectly up-regulate local vaginal cellular immunity prior to infection, enabling a rapid response to *C. albicans* and provide enhanced protection within the vaginal microenvironment. The up-regulation of IL-2, IL-4, and IL-17 with a reduced Th1/Th2 ratio was observed following treatment with rhIFNα-2b after *C. albicans* infection, which appeared to promote a Th17-biased response. Our data are in line with the previous finding that rhIFNα-2b initiates an early Th17-type innate immune response in VECs following *C. albicans* infection through promoting the production of granulocyte colony-stimulating factor, antimicrobial peptides, CXC chemokines, and recruiting neutrophil granulocytes during inflammation (Ouyang et al., 2008).

To date, Igs have been found to be produced by only differentiated B lymphocytes. However, recent evidence has demonstrated that non-B cancer cells and normal non-B cells are capable of producing Igs (Qiu et al., 2013). In addition, our previous research has also confirmed that normal VECs can secrete a type of functional non-B IgG rather than sIgA, challenging the classical concept that B cells are the only source of Ig (Li et al., 2016). Our study also confirms that non-B IgG expressed by VECs can be up-regulated following treatment with rhIFNα-2b alone. This form of IgG appears to be involved in the innate immune response of the vagina against mycotic infections, similar to the traditional IgG expressed by B cells, by recognizing foreign pathogens, neutralizing them directly, or activating other immune cells for their removal (Jiang et al., 2015). However, a reduction in IgG production was observed following rhIFNα-2b treatment post-*C. albicans* infection, which is interpreted as being due to decreased production by the VECs. Further studies should ascertain if vaginal epithelial-derived IgG participates in the local mucosal immunity of the vagina.

In this study, we used SEM to investigate the interaction between *C. albicans* and VECs. Our data suggest that *C. albicans* invades VECs via the induction of endocytosis and active penetration (Dalle et al., 2010). When treated with rhIFNα-2b for 24 h compared to infectious conditions, fungal viability appears to decline significantly as denoted by decreased *C. albicans* adhesion, invasion, and cellular injury. Furthermore, treated epithelial cells gradually become more thallus and completely stretched, exhibiting filopodia fusion and the production of a lamellipodium. The lamellipodium is a cytoskeletal protein actin projection on the leading edge of the cell, representing the mobility and the ability to repair from injury (Liu et al., 2015).

## Conclusion

Our data suggest an important protective role of type I IFN responses in the protective host response against yeast via: (1) stimulating the production of various functional cytokines by up-regulating IL-2 related cellular immunity and the IL-17 pathway; (2) increasing the secretion of vaginal epithelial-derived IgG, regulating antibody-mediated protection which plays an indispensable role in mucosal immunity against vaginitis; (3) effectively inhibiting the adhesion, hyphal formation, and proliferation of *C. albicans*; and (4) enhancing the mobility and the repair ability of the VECs. Further studies should explore the various molecular mechanisms and signaling pathways in the type I IFN responses against yeast.

## Author Contributions

The main experimental conception and design: ZL; Performed the experiments: TL and XN; Analyzed the data and contributed reagents: TL, XN, and ZL; Writing the manuscript: TL and XN. All the authors approved the final version.

## Conflict of Interest Statement

The authors declare that the research was conducted in the absence of any commercial or financial relationships that could be construed as a potential conflict of interest.

## Abbreviations

ANOVA: one-way analysis of variance
ELISA: enzyme-linked immunosorbent assay
FMF: familial Mediterranean fever
IC_10_: the 10% inhibition concentration
IFN: interferon
K-SFM: keratinocyte-serum free medium
MS: multiple sclerosis
PBS: phosphate-buffered saline
rhIFNα-2b: recombinant human IFNα-2b
SDA: Sabouraud’s dextrose agar
SEM: scanning electron microscopy
sIgA: secretory IgA
TLR: toll-like receptor
VECs: vaginal epithelial cells
VVC: vulvovaginal candidiasis
YPD: yeast peptone-dextrose.
